# Clonal integration and *Bacillus subtilis* modulate *Glechoma longituba* performance and soil microbial communities

**DOI:** 10.1371/journal.pone.0325605

**Published:** 2025-06-16

**Authors:** Bing-Nan Zhao, Xiao-Gai Wang, Rui Zhang, Xue-Ge He, Chao Si

**Affiliations:** 1 School of Life Science and Engineering, Handan University, Handan, China; 2 College of Life Sciences, Hebei University, Baoding, China; Graphic Era Institute of Technology: Graphic Era Deemed to be University, INDIA

## Abstract

Many medicinal plants exhibit clonality, but the impact of clonal integration and its interaction with exogenous microbial agents on these plants remains unknown. In order to investigate this, we conducted a greenhouse experiment using *Glechoma longituba*, a common clonal medicinal herb. Pairs of connected ramets were grown in the two adjacent pots, with one pot containing basal (relatively older) ramets treated with or without *Bacillus subtilis* agent and the other pot containing apical (relatively younger) ramets without *B. subtilis* agent treatment, the connection between basal and apical ramets were either left intact or severed. Clonal integration reduced the growth of basal ramets, but increased the apical ramet growth. *B. subtilis* agent primarily affected the root-shoot ratio of both basal and apical ramets as well as the whole fragments. Furthermore, it exhibited a significant interaction with clonal integration in affecting the root-shoot ratio of basal ramets and whole plant fragments. Addition of *B. subtilis* reduced the content of total flavonoids and chlorogenic acid in basal portions and chlorogenic acid at the whole fragment level. Clonal integration and *B. subtilis* agent significantly changed the composition of soil fungal communities of basal portions and bacterial communities of apical portions. The fungal composition of basal portions responded reciprocally to clonal integration and *B. subtilis*, with a significant increase in the relative abundance of Basidiomycota and a decrease in Ascomycota under clonal integration, whereas the effect of *B. subtilis* was opposite. *B. subtilis* significantly increased fungal diversity in basal portions while decreasing bacterial diversity in apical portions under clonal integration. However, neither clonal integration nor *B. subtilis* has showed a positive effect on the overall growth and quality of *G. longituba*. These findings provide valuable insights into its role in scientific cultivation and management of the clonal medicinal plants in the practical production.

## Introduction

Clonal integration is a crucial characteristic of clonal plants, realizing resource sharing among interconnected ramets [1–2]. Numerous studies have demonstrated that older ramets can effectively transfer resources to younger ones, thereby promoting their successful establishment and growth [1,3–10]. Moreover, ramets rooted under stressful or resource-poor conditions can acquire resources from connected ramets rooted in more favorable environments to enhance their survival via clonal integration [7,11,12].

Medicinal plants serve as a valuable natural resource for medicine, playing a pivotal role in both traditional Chinese medicine and modern pharmaceutical systems [13–15]. Enhancing the yield and quality of medicinal plants is crucial to meet the growing medical demands and ensure a stable supply of medicinal herbs products [16–18]. Notably, a large number of medicinal plants exhibit clonality, such as *Glechoma longituba*, *Duchesnea indica*, and *Mentha canadensis* [19–21]. These plants are rich in bioactive constituent, including flavonoids, phenolic acids, and terpenoids, and demonstrate pharmacological activities, such as antibacterial, antioxidant, choleretic, and diuretic effects [19–21]. However, very few studies have investigated the effects of clonal integration on the bioactive constituents of medicinal clonal plants, despite its potential to provide a theoretical basis for enhancing the yield and quality of these plants.

For clonal medicinal plants, clonal integration may influence growth performance and physiological processes by modulating resource allocation among interconnected ramets, thereby altering the accumulation of bioactive constituents [22–24]. Moreover, biotic stimuli such as microbial agents, are frequently utilized in agricultural practices to enhance soil microecology and promote plant growth [25–29]. These microbial agents can directly affect metabolic pathways by regulating root nutrient uptake (e.g., phosphorus and nitrogen) or indirectly stimulate the production of defense-related metabolites, such as terpenoids and phenolic compounds, through induced systemic resistance mechanisms [25–29]. Nevertheless, it remains unclear whether microbial agents can influence medicinal plants through clonal integration.

We conducted a greenhouse experiment to investigate the effects of clonal integration and *Bacillus subtilis* agent on the growth and bioactive constituent accumulation of *G. longituba*, a commonly used medicinal plant in China. Additionally, considering the intimate association between soil microorganisms and plants, we also investigated the effect of clonal integration and *B. subtilis* agent on the soil microbial communities in the root zone of *G. longituba*. We planted pairs of interconnected ramets of *G. longituba* in two adjacent pots, with the with or without the addition of *B. subtilis* agent into the soil where the basal (relatively older) ramets were rooted, while no *B. subtilis* agent was added into the soil where the apical (relatively younger) ramets were rooted. Furthermore, the stolons between the basal ramets and apical ramets were left intact or severed. Specifically, we aimed to test the following hypotheses: (i) clonal integration can affect the growth, bioactive constituent contents, and root zone microbial communities not only in basal and apical ramets but also throughout whole fragments; (ii) exogenous *B. subtilis* agent added to the basal ramets can affect the growth, bioactive constituent contents, and root zone microbial communities of the apical ramets through clonal integration.

## Materials and methods

### Plant species

*Glechoma longituba* is a clonal medicinal herb belonging to the Lamiaceae family [30–32]. It is abundant in flavonoids, chlorogenic acid, and other bioactive constituents [33–35]. The species produced a monopodial stolons with nodes that have the potential to generate ramets [36–38]. Each ramet bears two opposite leaves, which are heart-shaped or nearly kidney-shaped and covered with pubescence [12,39,40]. Every leaf axil contains one bud, which has the potential to develop into a secondary stolon [41,42]. It is widely distributed except in Qinghai, Gansu, Xinjiang, and Tibet in China [42,43]. The experimental plant materials were purchased from a commercial supplier located in Shanghai. Prior to the commencement of the experiment, the plants were cultivated in a greenhouse (36°34′N, 114°29′E) situated at Handan University in Handan City of Hebei Province, China.

On 11 May 2022, more than 100 ramets (consist one node and a pair of leaves) were cut from the stock plants cultivated in the greenhouse, as previously described, to prepare the plant materials required for subsequent experiments. These ramets were cultivated in seed tray filled with a substrate composed of vermiculite (particle size was 1–2 mm) and potting soil (pH value: 5.5–6.5, organic matter: > 45%; Jiqing Biotechnology Co. LTD, China) at a volume ratio of 1:1.

### Experimental design

The experiment employed a full factorial design, comprising two microbial agent treatments (with vs. without) and two stolon treatments (left intact vs. severed), resulting a total of four treatments (S1 Fig). On 5 June 2022, 20 well-developed fragments of uniform size (each consisting of 3–5 new ramets and an apex) were selected from the plant cultivated as the experimental material described earlier for use in this experiment. The three relatively older ramets referred to as the basal portion of the whole fragment, were rooted in the center of the pot (13 cm in diameter and 10.5 cm in height). The apex and its adjacent ramets, referred to as the apical portion, were allowed to root separately in another pot with the same size. Each pot was filled with a mixture of potting soil (7.9 g total N kg^-1^ and 224.7 g total C kg^-1^; Dewoduo Fertilizer Co., China) and locally collected topsoil (0.83 g total N kg^-1^ and 20.37 g total C kg^-1^) at a volume ratio of 1:1. On 15 June 2022, after confirming successful colonization of all plants, ten pairs of *G. longituba* were randomly selected for severing treatment by cutting the stolon that connected the basal and apical portions at their midpoint to made them to independent portions. The stolons of the rest plants were left intact, that is the basal and apical portions were interconnected.

In the treatments with the addition of microbial agent, 50 mL solution of *B. subtilis* agent with a concentration of 2 g L^-1^ was introduced into the soil where the basal portions were rooted every two weeks. The *B. subtilis* agent (Shandong, Tianguanjia, Biological Techonology Co., Ltd) used in this experiment was in the form of wettable powder, with a colony-forming unit (CFU) count of 2 × 10^12^ g^-1^. Simultaneously, 50 mL water was added to the soil where apical portions were rooted. All pots were randomly placed on a bench within the same greenhouse for material cultivation. The newly produced ramets from each portion were allowed to root in their respective original pots.

The experiment lasted until 20 July 2022. During the experiment, the mean air temperature and humidity in the greenhouse were measured every 2 hours by a temperature logger, with averages of 28.9°C and 53.1%, respectively. Throughout the experimental period, we maintained soil moisture in all pots by watering them based on the prevailing soil conditions.

### Measurements

At harvest, we meticulously removed the soil matrix adhered to the roots of *G. longituba* and quantified both ramet number and total stolon length. Subsequently, the plants were partitioned into leaves, stolons, and roots for thorough drying at 70^o^C until reaching a constant weight before being weighed. Additionally, we calculated the specific stolon length (total stolon mass/total stolon length) as well as the root-shoot ratio [root mass/ (leaf mass + stolon mass)]. The determination of total flavonoids and chlorogenic acid content was conducted using spectrophotometry with slight modifications to the method described by Li et al. [44] and Liu et al. [45].

We collected the soil from each pot and carefully removed visible debris, dirt, and plant roots. The soil samples were then fully mixed and stored at -40^o^C for subsequent analysis of the soil microbial communities. The total genomic DNA was extracted from the soil using the FastDNA SPIN kit (MP Biomedicals, USA) according to the manufacturer’s instructions. For bacteria, the V4 region of the bacterial 16S rRNA gene was amplified using the primer pair 515F (5’-GTGYCAGCMGCCGCGGTAA-3’) and 806R (5’-GACTACNVGGGTWTCTAAT-3’) [46]. For fungi, the ITS1 region of the fungal internal transcriptional spacer (ITS) was amplified with the primer pair ITS 1f (5’-CTTGGTCATTTAGAGGAAGTAA-3’) and ITS 2 (5’-GCTGGTTCTTCATCGATG C-3’) [46]. The end-paired sequencing of DNA fragments was performed on the Illumina Novaseq platform, provided by Shanghai Personal Biotechnology Co., LTD., Shanghai, China.

### Data analysis

Analysis of variance (ANOVA) was employed to examine the effects of clonal integration and *B. subtilis* agent on a series of measures about plant growth, bioactive constituents and soil microbial communities in this experiment. Before analysis, all data were checked for homoscedasticity and transformed as needed to improve homoscedasticity. The specific data transformation method are presented in the tables. All statistical analyses were conducted using SPSS 22.0 (IBM, Inc, ArInonk, NY, USA). In one of the experimental replicates where stolons were left intact without *B. subtilis* agent treatment, chlorogenic acid and total flavonoid content measurements could not be obtained due to insufficient sample amount; therefore these samples were excluded from further analyses.

In the bioinformatics analyses, the raw sequence data was filtered and initially processed using QIIME2 [47]. The Dada2 method was used to clip the bridge subsequences and primers from the original reads, eliminating low-quality sequences, chimeras, denoising, and splicing steps. Bacterial taxonomies were assigned by comparison with the Silva database [48], while fungal taxonomies were assigned by the UNITE database [49].

The alpha diversity indexes of bacterial and fungal communities, including Chao1, Shannon, Simpson, and Observed species, were subjected to statistical analysis using QIIME2. Additionally, the relative abundance of dominant bacterial taxa was assessed. Nonmetric multiple-dimensional scaling (NMDS) analysis was employed to visualize the beta diversity index across various treatments.

## Results

### Effects on growth and morphology

**Fig 1:**
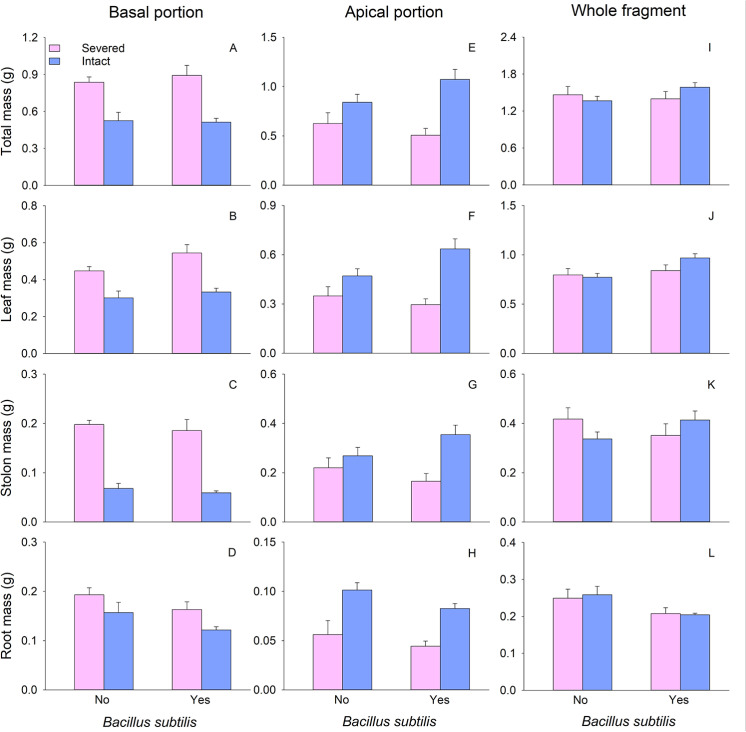
Effects of clonal integration and *Bacillus subtilis* on total mass (A, E, I), leaf biomass (B, F, J), stolon biomass (C, G, K), and root biomass (D, H, L) of the basal, apical portion, and whole fragment of *Glechoma longituba.* Bars and vertical lines represent mean and SE (n = 5).

**Fig 2:**
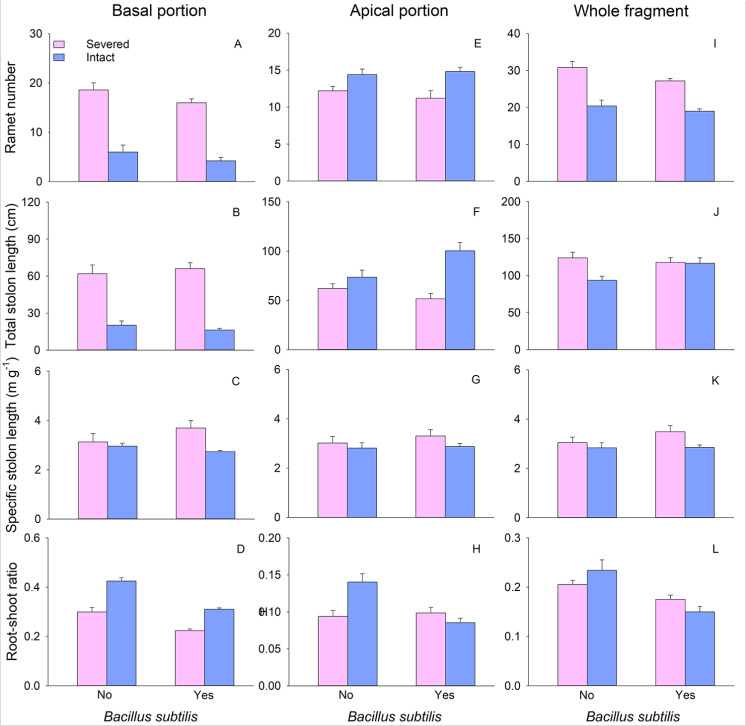
Effects of clonal integration and *Bacillus subtilis* on ramet number (A, E, I), total stolon length (B, F, J), specific stolon length (C, G, K), and root-shoot ratio (D, H, L) of the basal, apical portion, and whole fragment of *Glechoma longituba.* Bars and vertical lines represent mean and SE (n = 5).

The biomass (total mass, leaf mass, stolon mass, and root mass), ramet number, and total stolon length of basal portions of *G. longituba* were less when stolons left intact compared to when they were severed (Table 1; Figs 1A–1D, 2A and 2B). However, root-shoot ratio significantly increased when stolons were left intact (Table 1; Fig 2D). Clonal integration did not exert any effect on specific stolon length (Table 1). Only the root-shoot ratio of basal portions was affected by *B. subtilis* agent (Table 1). Moreover, the presence of *B. subtilis* agent resulted in a reduction in the root-shoot ratio (Fig 2D). The interaction between clonal integration and *B. subtilis* agent had no effect on any measures of plant growth or morphology in the basal portion (Table 1).

**Table 1:** Analysis of variance of the effects of clonal integration, *Bacillus subtilis*, and their interaction on growth and morphology of the basal portion, the apical portion and the whole fragment of *Glechoma longituba.*

Variable	Integration (I)		*Bacillus subtilis* (B)		I × B	
	F_1,16_	*P*	F_1,16_	*P*	F_1,16_	*P*
**Basal portion**						
Total mass ^a^	**34.1**	**< 0.001**	0.1	0.762	0.1	0.789
Leaf mass ^b^	**29.7**	**< 0.001**	3.6	0.074	0.5	0.498
Stolon mass ^a^	**99.3**	**< 0.001**	0.7	0.421	< 0.1	0.996
Root mass ^b^	**6.8**	**0.019**	4.5	0.051	< 0.1	0.827
Ramet number	**114.5**	**< 0.001**	3.7	0.072	0.1	0.730
Total stolon length ^b^	**110.2**	**< 0.001**	< 0.1	0.888	1.0	0.332
Specific stolon length ^a^	4.0	0.062	0.5	0.510	2.6	0.125
Root to shoot ratio ^b^	**81.5**	**< 0.001**	**63.2**	**< 0.001**	0.9	0.347
**Apical portion**						
Total mass ^b^	**18.6**	**0.001**	0.2	0.698	3.3	0.088
Leaf mass ^b^	**20.0**	**< 0.001**	0.8	0.398	4.2	0.057
Stolon mass ^b^	**11.1**	**0.004**	< 0.1	0.834	3.7	0.074
Root mass	**22.2**	**< 0.001**	3.0	0.103	0.2	0.698
Ramet number	**14.8**	**0.001**	0.2	0.696	0.9	0.368
Total stolon length ^b^	**20.1**	**< 0.001**	0.4	0.544	**7.7**	**0.014**
Specific stolon length	2.0	0.172	0.6	0.438	0.3	0.616
Root to shoot ratio ^a^	2.9	0.109	**8.2**	**0.011**	**12.3**	**0.003**
**Whole clonal fragment**						
Total mass ^a^	0.3	0.590	0.6	0.456	1.9	0.187
Leaf mass	1.0	0.335	**5.3**	**0.035**	2.1	0.165
Stolon mass ^b^	< 0.1	0.862	< 0.1	0.908	3.4	0.084
Root mass ^a^	< 0.1	0.840	**6.3**	**0.023**	0.1	0.786
Ramet number	**57.3**	**< 0.001**	4.1	0.059	0.8	0.384
Total stolon length ^a^	**5.6**	**0.030**	1.9	0.192	**4.8**	**0.044**
Specific stolon length ^a^	3.9	0.066	1.2	0.289	0.8	0.372
Root to shoot ratio ^a^	0.1	0.742	**21.2**	**< 0.001**	**4.7**	**0.046**

^a^Natural log transformation.

^b^Square root transformation. Degree of freedom (subscript for “F”), F and *P* values are given. Values are in bold when *P* < 0.05.

The biomass, ramet number, and total stolon length of apical portions were greater when stolons were left intact compared to when they were severed (Table 1; Figs 1E–1H, 2E and 2F). There was no different in specific stolon length and root-shoot ratio between the two stolon treatments (Table 1). The presence of *B. subtilis* agent significantly reduced the root-shoot ratio but had no effect on other measures of apical portions (Table 1; Fig 2H). The interaction between clonal integration and *B. subtilis* significantly affected total stolon length and root-shoot ratio (Table 1). Clonal integration increased total stolon length with the addition of *B. subtilis* (Fig 2F). When stolons were severed, *B. subtilis* significantly increased the root-shoot ratio, whereas this effect was not observed when stolons were left intact (Fig 2H).

At the whole fragment level, there were no significant differences in biomass, specific stolon length, and root-shoot ratio between the two stolon treatments (Table 1). However, intact stolons treatment exhibited lower ramet number and total stolon length compared to severed stolons treatment (Table 1; Fig 2I and 2J). Leaf mass was higher in the treatment with *B. subtilis* while root mass and root-shoot ratio were lower compared to treatments without *B. subtilis* (Table 1; Figs 1J, 1L and 2L). The interaction between clonal integration and *B. subtilis* significantly affected total stolon length and root-shoot ratio (Table 1). Specifically, when stolons were severed, *B. subtilis* resulted in a significant decrease in total stolon length and root-shoot ratio, when stolons were left intact, *B. subtilis* significantly increased the total stolon length but significantly decreased the root-shoot ratio (Fig 2J and 2L).

### Effects on total flavonoids and chlorogenic acid

**Fig 3:**
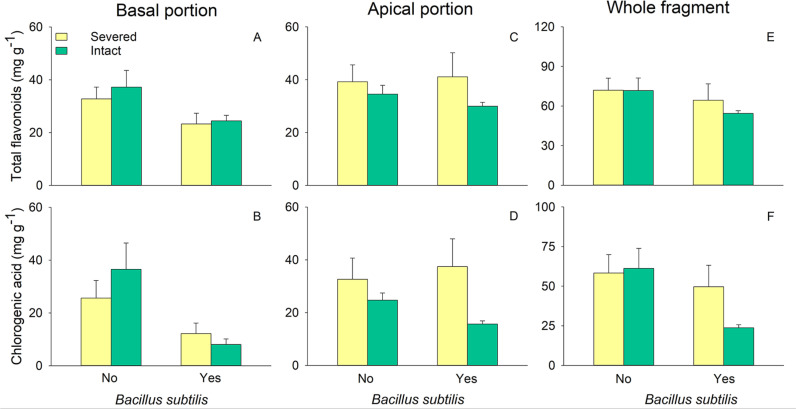
Effects of clonal integration and *Bacillus subtilis* on total flavonoids (A, C, E) and chlorogenic acid (B, D, F) of the basal, apical portion, and whole fragment of *Glechoma longituba.* Bars and vertical lines represent mean and SE (n = 5 except n = 4 in one treatment; details are described in the data analysis section).

In the basal portions, no significant differences were observed in content of total flavonoids or chlorogenic acid between the two stolon treatments (Table 2). The addition of *B. subtilis* agent resulted in lower levels of total flavonoids and chlorogenic acid in the basal portions compared to those without the agent (Table 2; Fig 3A and 3B). The interaction between stolon treatment and *B. subtilis* agent did not significantly affect the content of total flavonoids or chlorogenic acid in the basal portions (Table 2).

**Table 2:** Analysis of variance of the effects of clonal integration, *Bacillus subtilis*, and their interaction on total flavonoids and chlorogenic acid of the basal portion, the apical portion, and the whole fragment of *Glechoma longituba.*

Variable	Integration (I)		*Bacillus subtilis* (B)		I × B	
	F_1,15_	*P*	F_1,15_	*P*	F_1,15_	*P*
**Basal portion**						
Total flavonoids ^a^	0.5	0.485	**6.6**	**0.022**	< 0.1	0.925
Chlorogenic acid ^a^	< 0.1	0.968	**15.2**	**0.001**	1.4	0.256
**Apical portion**						
Total flavonoids ^a^	0.6	0.433	0.3	0.617	0.2	0.684
Chlorogenic acid ^a^	3.0	0.103	0.6	0.458	1.1	0.304
**Whole clonal fragment**						
Total flavonoids	0.3	0.584	1.9	0.192	0.3	0.602
Chlorogenic acid ^a^	0.9	0.363	**5.3**	**0.037**	1.8	0.204

^a^Natural log transformation. Degree of freedom (subscript for “F”), F and *P* values are given. Values are in bold when *P* < 0.05.

In the apical portions, neither stolon treatment, *B. subtilis* agent, nor their interaction had any significant effect on the content of total flavonoids or chlorogenic acid (Table 2).

At the whole fragment level, the addition of *B. subtilis* agent significantly reduced the content of chlorogenic acid (Table 2; Fig 3F). There was no significant effect of stolon, *B. subtilis* or their interaction on these active constituents (Table 2).

### Effects on soil microbial communities

**Fig 4:**
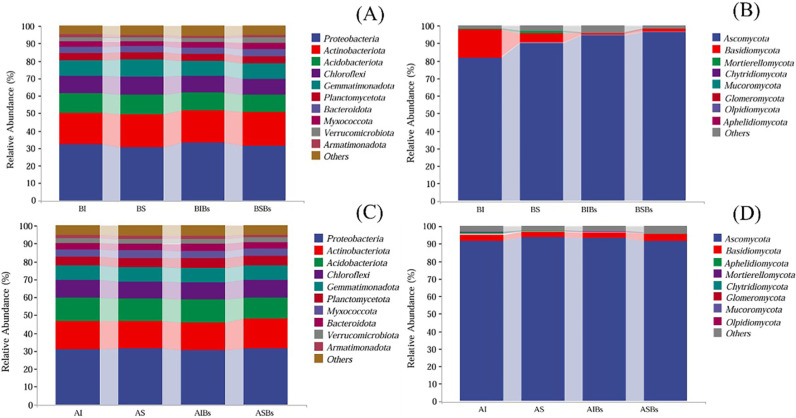
The composition of bacterial (A) and fungal (B) communities of the basal portion and bacterial (C) and fungal (D) communities of the apical portion under different treatments (n = 3). BI: Basal soils in intact stolon treatment without *Bacillus subtilis*; BS: Basal soils in severed stolon treatment without *B. subtilis*; BIBs: Basal soils in intact stolon treatment with *B. subtilis*; BSBs: Basal soils in severed stolon treatment with *B. subtilis*; AI: Apical soils in intact stolon treatment without *B. subtilis*; AS: Apical soils in severed stolon treatment without *B. subtilis*; AIBs: Apical soils in intact stolon treatment with *B. subtilis*; ASBs: Apical soils in severed stolon treatment with *B. subtilis*.

A total of 2,266,800 high-quality reads (bacterial 16S rRNA gene) and 2,381,809 high-quality reads (fungal ITS1 region) were retained after sequencing and quality filtering from all samples. At the phyla level, Proteobacteria, Actinobacteriota, Acidobacteriota, Chloroflexi, Gemmatimonadota, Planctomycetota, Bacteroidota, Myxococcota, Verrucomicrobiota and Armatimonadota were identified as the predominant bacterial taxa accounting for 94.0% to 94.8% under different treatments (Fig 4A and 4C). Clonal integration and *B. subtilis* did not significantly affect the composition of bacterial communities in the root zone soil of basal and apical portions; however, while their interaction had a significant effect on the relative abundance of Verrucomicrobiota of basal portions (S1 and S2 Tables). *B. subtilis* decreased the relative abundance of Verrucomicrobiota when the stolon was left intact, but increased it when the stolon was severed (Fig 4A). Regarding fungi composition analysis, the most predominant fungal taxa were Ascomycota and Basidiomycota which accounted for 95.1% to 98.1% under different treatments (Fig 4B and 4D). Clonal integration significantly increased the relative abundance of Basidiomycota, but significantly decreased the relative abundance of Ascomycota in root zone soil of basal portions (S1 Table; Fig 4B). *B. subtilis* significantly increased the relative abundance of Ascomycota but significantly decreased the relative abundance of Basidiomycota and Chytridiomycota (S1 Table; Fig 4B). Moreover, the interaction between clonal integration and *B. subtilis* significantly affected the relative abundance of Chytridiomycota of apical portions (S2 Table). *B. subtilis* increased the relative abundance of Chytridiomycota when the stolon was left intact (Fig 4D).

**Fig 5:**
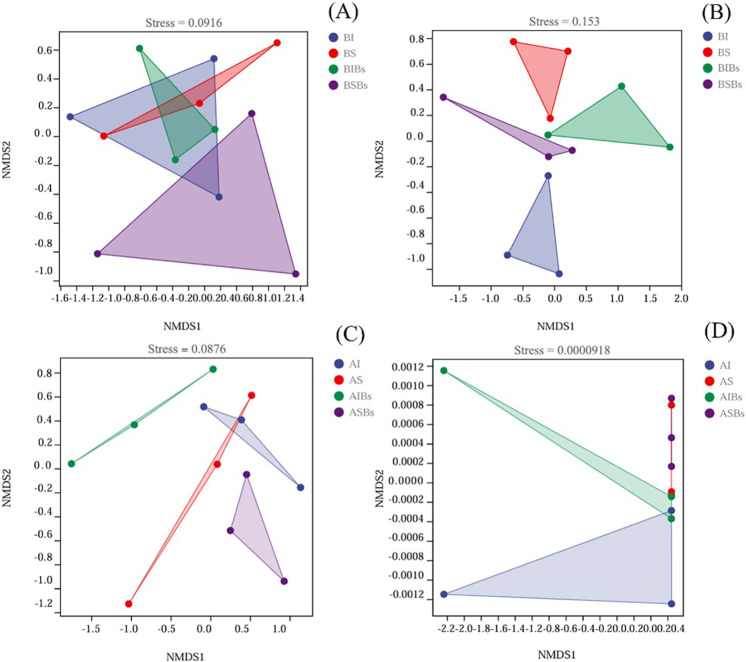
The NMDS plots of bacterial (A) and fungal (B) communities of the basal portion and bacterial (C) and fungal (D) communities of the apical portion based on Bray-Curits distance (n = 3). BI: Basal soils in intact stolon treatment without *Bacillus subtilis*; BS: Basal soils in severed stolon treatment without *B. subtilis*; BIBs: Basal soils in intact stolon treatment with *B. subtilis*; BSBs: Basal soils in severed stolon treatment with *B. subtilis*; AI: Apical soils in intact stolon treatment without *B. subtilis*; AS: Apical soils in severed stolon treatment without *B. subtilis*; AIBs: Apical soils in intact stolon treatment with *B. subtilis*; ASBs: Apical soils in severed stolon treatment with *B. subtilis*.

The NMDS analysis results showed that bacterial community composition of basal did not show significant differences between the different treatments (Fig 5A). Furthermore, the two-dimensional plots clearly demonstrated that the fungal communities of basal soils in intact stolon treatment without *B. subtilis* (BI) and basal soils in severed stolon treatment without *B. subtilis* (BS) were distinctly separated along the second axis, with a clear separation between presence or absence of *B. subtilis* agent, indicating substantial alterations to soil fungal communities of basal portions due to clonal integration and *B. subtilis* (Fig 5B). Additionally, there were variations observed in the distribution patterns of bacterial and fungal communities between basal and apical portions. The bacterial community composition of apical soils showed significant differences along the first axis of NMDS plots for apical soil in intact stolon treatment without *B. subtilis* (AI) and apical soil in intact stolon treatment with *B. subtilis* (AIBs), while there was also a clear separation in bacterial community composition between AI and apical soil in severed stolon treatment without *B. subtilis* (AS) (Fig 5C). On the other hand, no significant differences were found in fungal community composition among different treatments for apical portions (Fig 5D).

**Fig 6:**
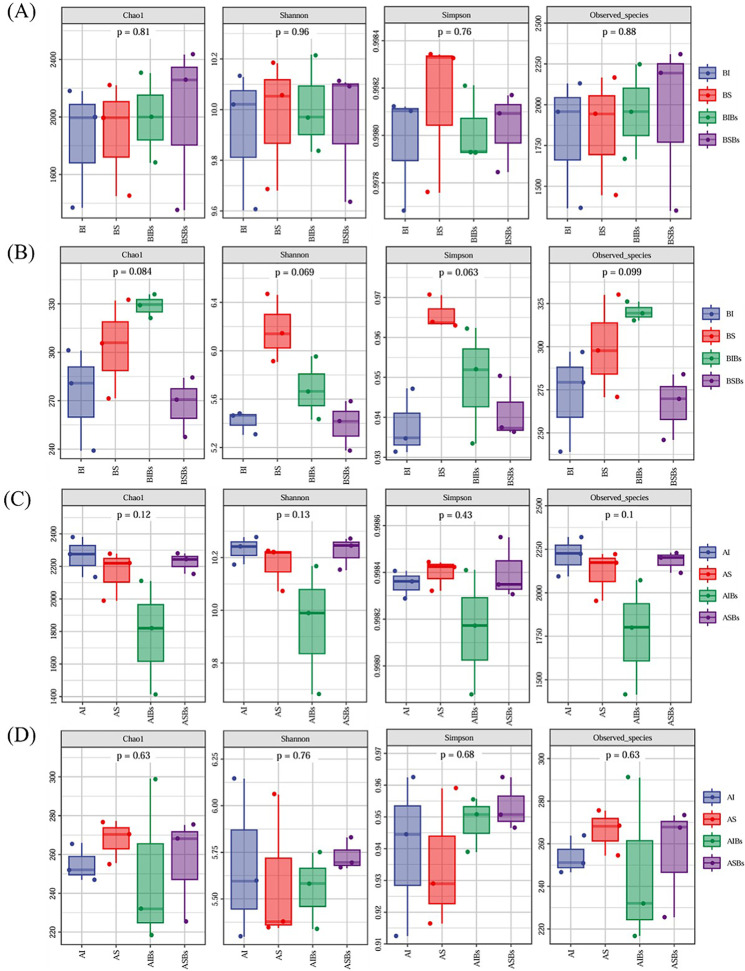
Microbial alpha diversity of bacterial (A) and fungal (B) communities of the basal portion and bacterial (C) and fungal (D) communities of the apical portion under different treatment (n = 3). BI: Basal soils in intact stolon treatment without *Bacillus subtilis*; BS: Basal soils in severed stolon treatment without *B. subtilis*; BIBs: Basal soils in intact stolon treatment with *B. subtilis*; BSBs: Basal soils in severed stolon treatment with *B. subtilis*; AI: Apical soils in intact stolon treatment without *B. subtilis*; AS: Apical soils in severed stolon treatment without *B. subtilis*; AIBs: Apical soils in intact stolon treatment with *B. subtilis*; ASBs: Apical soils in severed stolon treatment with *B. subtilis*.

Clonal integration and *B. subtilis* did not significant affect bacterial alpha diversity in root zone soil of basal and apical portions of *G. longituba* (S3 Table). However, the interaction between clonal integration and *B. subtilis* had a significant effect on the bacterial alpha diversity of apical portions (S3 Table). When the stolon was left intact, *B. subtilis* reduced the bacterial alpha diversity of apical portions (S3 Table; Fig 6C). Clonal integration and *B. subtilis* did not have a significant effect on fungal alpha diversity in root zone soil of basal and apical portions, but their interaction significantly affected fungal alpha diversity in root zone soil of basal portions (S3 Table). When the stolon was left intact, *B. subtilis* increased fungal alpha diversity (Fig 6B).

## Discussion

### Responses of growth and morphology

Clonal integration is a crucial trait that realizes resource transfer from basal ramets to the connected apical ramets, thereby promoting their survival and growth [43,50–52]. This resource transfer establishes a cost-benefit relationship between basal and apical ramets [6,12,53]. However, previous studies have reported inconsistent findings regarding whether clonal integration leads to reduced growth in basal ramets [3,4,6–9,12,53]. In some studies, despite providing resources to apical ramets, basal ramets remained unaffected and even benefited from the compensatory responses to the stress experienced by apical ramets [3,8,9,54]. Conversely, other studies indicated that basal ramets might suffer growth reductions due to resource sharing with apical ramets [6,7,53]. At the whole plant level, the benefits of clonal integration depended on whether the cost incurred by basal ramets outweighs the advantages gained by apical ramets. In this experiment, intact stolons led to increased biomass accumulation, ramet number, and total stolon length in apical portions but resulted in decreased these measurements for basal portions. For whole fragments, maintaining intact stolons did not affect biomass but significantly reduced ramet number and total stolon length. These findings suggested that clonal integration enhanced the growth (measured as biomass), vegetative propagation potential (measured as ramet number), and expansion ability (measured as total stolon length) of apical portions of *G. longituba* due to resource provision by the basal portions. However, clonal integration may not promote biomass accumulation of the whole fragments of due to the equal trade-off between the benefits gained by apical portions and the costs incurred by basal portions, which is a finding consistent with our previous study [12]. Nevertheless, clonal integration exhibited a significantly negative effect on vegetative propagation and expansion ability, also aligning with our earlier finding [12]. Furthermore, clonal integration increases the root-shoot ratio of basal portions, suggesting an adaptive strategy that favors enhanced resource uptake such as water and nutrients through roots from soil for the benefit of both apical and basal portions growth [55–57].

Although numerous studies have demonstrated the positive impacts of exogenous microbial agents on plant growth [58,59], no such effects were observed in basal portions upon addition of the *B. subtilis* agent in this experiment. However, a significant effect of the *B. subtilis* agent on root-shoot ratio was detected in both basal and apical portions, as well as in the whole fragments. In basal portions, the exogenous *B. subtilis* agent resulted in a decrease in root-shoot ratio, implying its potential to enhance biomass allocation towards shoot development. Moreover, the interaction between the *B. subtilis* agent and stolon treatment affected biomass allocation in apical portions and whole fragments, suggesting that *B. subtilis* can increase their shoot mass allocation through clonal integration. Additionally, an intriguing finding from this study revealed that when stolons remained intact and *B. subtilis* agent was added to basal portions significantly increased total stolon length not only in apical portions but also throughout whole fragments alike which may further promote expansion of apical portions. This indicates that, although initially applied only to the basal portions, the effect of *B. subtilis* can propagate to the apical portions through clonal integration. Moreover, it may exhibit a potential to enhance the aboveground yield of *G. longituba* at the whole fragment level.

### Responses of total flavonoids and chlorogenic acid

Although the growth and morphology of both the basal and apical portions of *G. longituba* were significantly affected by the stolon treatment, no significant differences were observed in the content of two main active constituents-total flavonoids and chlorogenic acid-across different stolon treatments. This suggests that while the clonal integration has a positive on plant growth, its effect on regulating bioactive constituents is relatively limited. The total flavonoid and chlorogenic acid content in the basal portions, as well as the chlorogenic acid content in the whole fragment, exhibited a significant negative response to *B. subtilis*. Specifically, the addition of *B. subtilis* led to a decrease in these active constituents, which was observed not only in the basal portions but also verified in the whole fragment. This indicates that *B. subtilis* may inhibit the accumulation of these active constituents through certain mechanisms or alter metabolic pathways within the plant, resulting in reduced content. Based on this study, we are unable to fully explain the specific role of *B. subtilis* in regulating the bioactive constituents of *G. longituba*. Exogenous microbial agents are widely used in agriculture and horticulture and are generally recognized for promoting plant growth [58,59]. However, this study shows that the application of *B. subtilis* in *G. longituba* did not achieve the expected outcomes in terms of improving quality and even had a negative effect. Our findings highlight that different plants may respond differently to microbial agents, underscoring the importance of selecting appropriate microbial agents and application methods for specific plant species.

### Responses of soil microbial communities

Clonal integration can affect plant growth and rhizosphere soil nitrogen transformation by sharing resource among interconnected ramets, thereby influencing the composition of soil microorganisms [60]. Our findings demonstrated that both clonal integration and *B. subtilis* significantly affected the composition of soil fungal communities of basal portions and bacterial communities of apical portions. Furthermore, while neither clonal integration nor *B. subtilis* affect alpha diversity of soil bacterial and fungal in the root zone soil of *G. longituba*, their interaction significantly affected microbial diversity. Through clonal integration, *B. subtilis* significantly increased fungi diversity of basal portions but decreased bacteria diversity of apical portions; these results indicated that bacterial and fungal communities exhibit different responses to clonal integration and *B. subtilis* agent between basal and apical portions. One potential explanation is that fungi may be more susceptible to the influence of plant root exudates, while bacteria may be more susceptible to the influence of the external microenvironment [61]. Clonal integration resulted in reduced growth of basal ramets and increased their total flavonoid and chlorogenic acid content, leading to changes in fungal community composition in root-zone soils. We hypothesized the total flavonoid and chlorogenic acid content in plants could significantly affect the composition of fungal communities in root zone soils. Previous studies have demonstrated that *B. subtilis* can improve soil environment and alter soil fungal community composition through metabolite secretion and release of signaling substances [62–64]. Additionally, our results confirmed that clonal integration can combined with *B. subtilis* modulate biomass allocation to roots in apical portions, as well as influence the bacterial communities within their root zone soils. Unfortunately, further investigation is needed to elucidate the underlying mechanism behind this influence. Furthermore, clonal integration and *B. subtilis* had significant effects on the abundance of Ascomycota and Basidiomycota in basal portions root zone soils, but they exerted opposite trends regarding their influence. This result further supported that *B. subtilis* agent diminished certain advantages derived from clonal integration in basal ramets.

## Conclusion

We conclude that *B. subtilis* enhances aboveground yield through clonal integration while suppressing the accumulation of flavonoids and chlorogenic acid. Its interaction with clonal integration reshapes soil microbial diversity and partially offsetting the effects of clonal integration via counteractive shifts in fungal composition. These findings establish a theoretical foundation for optimizing the “microbial-clonal integration regulation” cultivation model in medicinal plants agriculture. In future research, transcriptomics and metabolomics could be utilized to elucidate the mechanisms underlying the effects of *B. subtilis* and clonal integration, and field-scale validation experiments could be conducted to bridge the gap between theory and practical application.

## Supporting information

S1 Table Analysis of variance of the effects of clonal integration, *Bacillus subtilis*, and their interaction on composition of bacterial and fungal communities at phyla level in root zone soil of the basal portion of *Glechoma longituba.* (DOCX)

S2 Table Analysis of variance of the effects of clonal integration, *Bacillus subtilis*, and their interaction on composition of bacterial and fungal communities at phyla level in root zone soil of the apical portion of *Glechoma longituba.* (DOCX)

S3 Table Analysis of variance of the effects of clonal integration, *Bacillus subtilis*, and their interaction on bacterial and fungal alpha diversity in root zone soil of the basal portion and the apical portion of *Glechoma longituba.* (DOCX)

S1 Fig Schematic representation of the experimental design. Basal portions of *Glechoma longituba* were grown in soil with or without the addition of *Bacillus subtilis*, while apical portions were grown in soil without *B. subtilis*. The stolon between basal and apical portions were either left intact or severed.
(TIF)
