# Supplementary material for: Clonal integration and Bacillus subtilis modulate Glechoma longituba performance and soil microbial communities
Source: PLoS One. 2025 Jun 16;20(6):e0325605. doi: 10.1371/journal.pone.0325605 (PMC12169573; doi:10.1371/journal.pone.0325605)
Supplement: S1 Table — (DOCX) [file pone.0325605.s001.docx]

**S1Table Analysis of variance of the effects of clonal integration, *Bacillus subtilis*, and their interaction on composition of bacterial and fungal communities at phyla level in root zone soil of the basal portion of *Glechoma longituba*.**

| Variable |  | Integration (I) | |  | *Bacillus subtilis* (B) | |  | I × B | |
| --- | --- | --- | --- | --- | --- | --- | --- | --- | --- |
|  |  | F_1, 8_ | *P* |  | F_1, 8_ | *P* |  | F_1, 8_ | *P* |
| **Basal portion** | | | | | | | | | |
| *Bacterial communities* | | | | | | | | | |
| Proteobacteria |  | 4.2 | 0.075 |  | 1.4 | 0.278 |  | < 0.1 | 0.958 |
| Actinobacteriota ^a^ |  | 0.2 | 0.644 |  | 0.1 | 0.752 |  | < 0.1 | 0.821 |
| Acidobacteriota ^a^ |  | < 0.1 | 0.903 |  | 3.2 | 0.112 |  | 0.8 | 0.390 |
| Chloroflexi |  | < 0.1 | 0.825 |  | 2.9 | 0.129 |  | 0.5 | 0.485 |
| Gemmatimonadota ^a^ |  | 0.9 | 0.361 |  | 1.4 | 0.271 |  | 0.1 | 0.753 |
| Planctomycetota ^a^ |  | 0.2 | 0.664 |  | 0.5 | 0.512 |  | 0.1 | 0.746 |
| Myxococcota |  | < 0.1 | 0.860 |  | 4.0 | 0.081 |  | 0.6 | 0.454 |
| Bacteroidota ^a^ |  | 0.1 | 0.736 |  | 0.9 | 0.370 |  | 2.0 | 0.197 |
| Verrucomicrobiota ^a^ |  | 3.0 | 0.124 |  | 1.9 | 0.201 |  | **7.2** | **0.028** |
| Armatimonadota ^a^ |  | 1.7 | 0.228 |  | 2.8 | 0.132 |  | 0.1 | 0.780 |
|  | | | | | | | | | |
| *Fungal communities* | | | | | | | | | |
| Ascomycota |  | **8.2** | **0.021** |  | **32.5** | **< 0.001** |  | 2.6 | 0.143 |
| Basidiomycota ^a^ |  | **7.3** | **0.027** |  | **65.2** | **< 0.001** |  | 5.0 | 0.055 |
| Mortierellomycota ^a^ |  | 3.7 | 0.090 |  | 0.8 | 0.399 |  | 0.4 | 0.544 |
| Aphelidiomycota |  | 1.0 | 0.347 |  | 1.0 | 0.347 |  | 1.0 | 0.347 |
| Chytridiomycota ^b^ |  | 0.5 | 0.492 |  | **6.0** | **0.040** |  | 0.6 | 0.462 |
| Glomeromycota ^b^ |  | 1.4 | 0.266 |  | 1.2 | 0.298 |  | 1.3 | 0.289 |
| Mucoromycota |  | 0.6 | 0.446 |  | 0.7 | 0.425 |  | 1.5 | 0.252 |
| Olpidiomycota |  | 3.7 | 0.092 |  | 3.7 | 0.092 |  | 3.7 | 0.092 |

^a^ Natural log transformation. ^b^ Square root transformation. Degree of freedom (subscript for “F”), F and *P* values are given. Values are in bold when *P*＜0.05.
