# Supplementary material for: Clonal integration and Bacillus subtilis modulate Glechoma longituba performance and soil microbial communities
Source: PLoS One. 2025 Jun 16;20(6):e0325605. doi: 10.1371/journal.pone.0325605 (PMC12169573; doi:10.1371/journal.pone.0325605)
Supplement: S2 Table — (DOCX) [file pone.0325605.s002.docx]

**S2 Table Analysis of variance of the effects of clonal integration, *Bacillus subtilis*, and their interaction on composition of bacterial and fungal communities at phyla level in root zone soil of the apical portion of *Glechoma longituba*.**

| Variable |  | Integration (I) | |  | *Bacillus subtilis* (B) | |  | I × B | |
| --- | --- | --- | --- | --- | --- | --- | --- | --- | --- |
|  |  | F_1, 8_ | *P* |  | F_1, 8_ | *P* |  | F_1, 8_ | *P* |
| **Apical portion** | | | | | | | | | |
| *Bacterial community* | | | | | | | | | |
| Proteobacteria |  | 1.4 | 0.264 |  | 0.1 | 0.719 |  | 0.7 | 0.416 |
| Actinobacteriota |  | < 0.1 | 0.814 |  | 0.2 | 0.694 |  | 0.5 | 0.500 |
| Acidobacteriota ^a^ |  | 1.3 | 0.295 |  | 0.3 | 0.571 |  | 1.0 | 0.338 |
| Chloroflexi ^b^ |  | 0.1 | 0.735 |  | 0.1 | 0.733 |  | 3.4 | 0.102 |
| Gemmatimonadota ^a^ |  | < 0.1 | 0.763 |  | 0.2 | 0.661 |  | < 0.1 | 0.883 |
| Planctomycetota ^a^ |  | 0.9 | 0.371 |  | 0.6 | 0.479 |  | 0.2 | 0.689 |
| Myxococcota ^a^ |  | 0.1 | 0.732 |  | < 0.1 | 0.973 |  | 1.0 | 0.355 |
| Bacteroidota ^a^ |  | 1.5 | 0.257 |  | 3.3 | 0.106 |  | < 0.1 | 0.973 |
| Verrucomicrobiota |  | 1.8 | 0.222 |  | < 0.1 | 0.965 |  | 2.5 | 0.150 |
| Armatimonadota ^a^ |  | 1.1 | 0.331 |  | 2.1 | 0.188 |  | 0.9 | 0.369 |
|  | | | | | | | | | |
| *Fungal community* | | | | | | | | | |
| Ascomycota |  | < 0.1 | 0.970 |  | < 0.1 | 0.859 |  | 1.7 | 0.228 |
| Basidiomycota ^b^ |  | 0.7 | 0.416 |  | 0.1 | 0.710 |  | 0.2 | 0.639 |
| Mortierellomycota ^a^ |  | 0.4 | 0.558 |  | 0.6 | 0.447 |  | 1.5 | 0.250 |
| Aphelidiomycota |  | 2.8 | 0.133 |  | 2.5 | 0.150 |  | 1.3 | 0.292 |
| Chytridiomycota ^b^ |  | 1.1 | 0.321 |  | 0.1 | 0.744 |  | **5.8** | **0.042** |
| Glomeromycota ^b^ |  | 1.9 | 0.210 |  | 0.7 | 0.440 |  | < 0.1 | 0.823 |
| Mucoromycota |  | 1.0 | 0.347 |  | 1.0 | 0.347 |  | 1.0 | 0.347 |

^a^ Natural log transformation. ^b^ Square root transformation. Degree of freedom (subscript for “F”), F and *P* values are given. Values are in bold when *P*＜0.05.
