# Supplementary material for: Clonal integration and Bacillus subtilis modulate Glechoma longituba performance and soil microbial communities
Source: PLoS One. 2025 Jun 16;20(6):e0325605. doi: 10.1371/journal.pone.0325605 (PMC12169573; doi:10.1371/journal.pone.0325605)
Supplement: S3 Table — (DOCX) [file pone.0325605.s003.docx]

**S3 Table Analysis of variance of the effects of clonal integration, *Bacillus subtilis*, and their interaction on bacterial and fungal alpha diversity in root zone soil of the basal portion and the apical portion of *Glechoma longituba*.**

| Variable |  | Integration (I) | |  | *Bacillus subtilis* (B) | |  | I × B | |
| --- | --- | --- | --- | --- | --- | --- | --- | --- | --- |
|  |  | F_1, 8_ | *P* |  | F_1, 8_ | *P* |  | F_1, 8_ | *P* |
| **Basal portion** | | | | | | | | | |
| *Bacterial diversity* | | | | | | | | | |
| Chao1 |  | < 0.1 | 0.910 |  | 0.3 | 0.604 |  | < 0.1 | 0.968 |
| Observed species |  | < 0.1 | 0.954 |  | 0.3 | 0.626 |  | < 0.1 | 0.934 |
| Shannon |  | < 0.1 | 0.984 |  | < 0.1 | 0.834 |  | 0.2 | 0.702 |
| Simpson |  | 0.5 | 0.521 |  | < 0.1 | 0.848 |  | 0.3 | 0.575 |
|  | | | | | | | | | |
| *Fungal diversity* | | | | | | | | | |
| Chao1 |  | 1.3 | 0.288 |  | 0.5 | 0.505 |  | **10.6** | **0.012** |
| Observed species |  | 0.9 | 0.361 |  | 0.3 | 0.579 |  | **9.2** | **0.016** |
| Shannon ^a^ |  | 3.0 | 0.120 |  | 3.9 | 0.085 |  | **16.8** | **0.003** |
| Simpson |  | 3.3 | 0.105 |  | 1.4 | 0.268 |  | **10.7** | **0.011** |
|  | | | | | | | | | |
| **Apical portion**  *Bacterial diversity* | | | | | | | | | |
| Chao1 |  | 2.1 | 0.182 |  | 3.2 | 0.112 |  | **5.4** | **0.048** |
| Observed species |  | 2.1 | 0.182 |  | 3.0 | 0.119 |  | **5.5** | **0.047** |
| Shannon |  | 1.9 | 0.205 |  | 2.2 | 0.177 |  | 4.5 | 0.066 |
| Simpson |  | 2.7 | 0.141 |  | 1.2 | 0.311 |  | 1.3 | 0.287 |
|  | | | | | | | | | |
| *Fungal diversity* | | | | | | | | | |
| Chao1 ^a^ |  | 0.5 | 0.512 |  | 0.4 | 0.540 |  | < 0.1 | 0.901 |
| Observed species ^a^ |  | 0.6 | 0.460 |  | 0.5 | 0.495 |  | < 0.1 | 0.952 |
| Shannon ^a^ |  | < 0.1 | 0.805 |  | < 0.1 | 0.937 |  | 0.5 | 0.491 |
| Simpson |  | < 0.1 | 0.991 |  | 1.8 | 0.222 |  | 0.2 | 0.646 |

^a^ Natural log transformation. Degree of freedom (subscript for “F”), F and *P* values are given. Values are in bold when *P*＜0.05.
