# Supplementary figures and images for: Clonal integration and Bacillus subtilis modulate Glechoma longituba performance and soil microbial communities
Source: PLoS One. 2025 Jun 16;20(6):e0325605. doi: 10.1371/journal.pone.0325605 (PMC12169573; doi:10.1371/journal.pone.0325605)

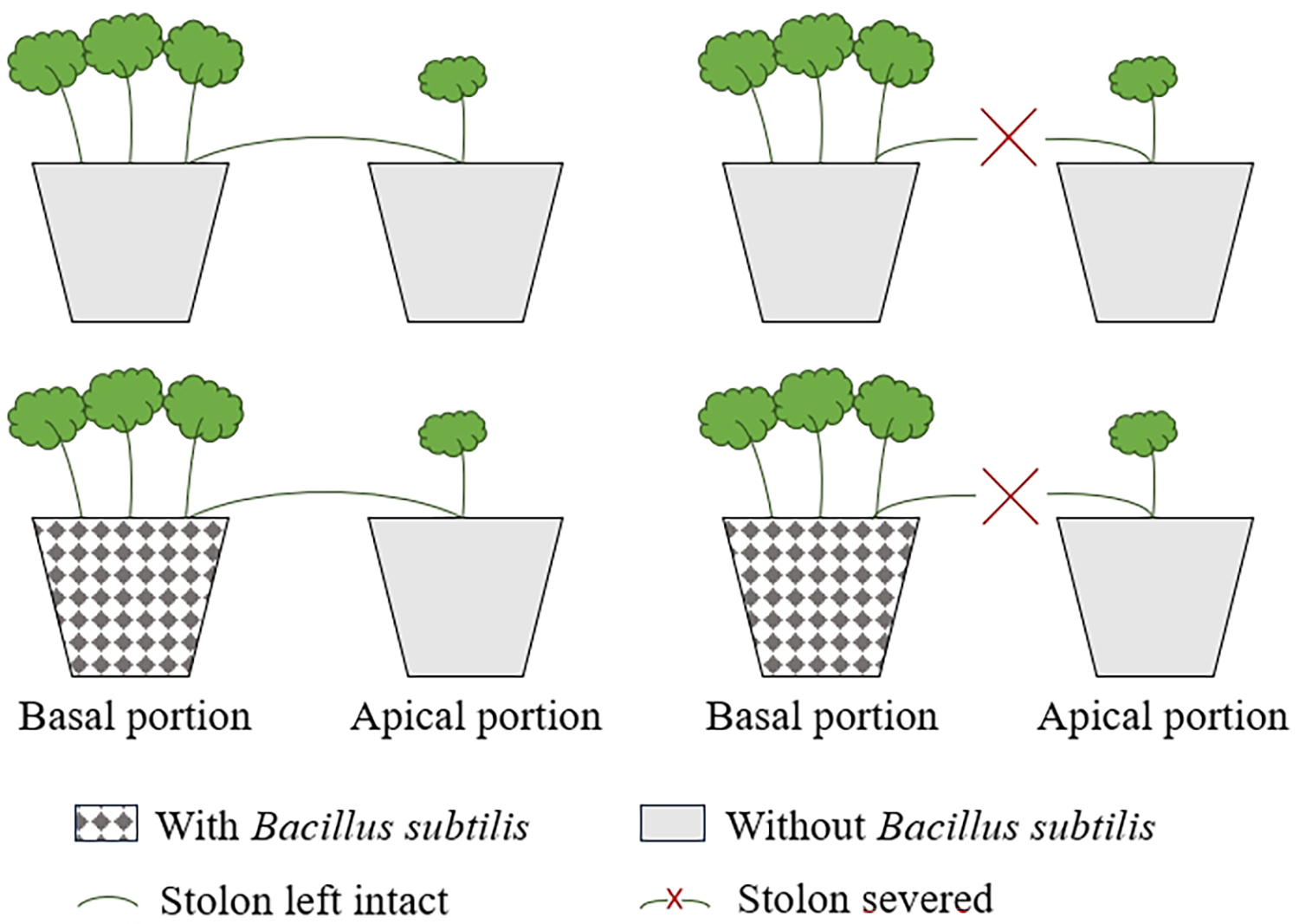

Supplement: S1 Fig — Basal portions of Glechoma longituba were grown in soil with or without the addition of Bacillus subtilis, while apical portions were grown in soil without B. subtilis. The stolon between basal and apical portions were either left intact or severed. (TIF) [file pone.0325605.s004.tif]
